# Reviewing Evidence for the Usefulness of Family Interventions for Depression During and After the COVID-19 Pandemic

**DOI:** 10.1192/j.eurpsy.2022.78

**Published:** 2022-09-01

**Authors:** G. Keitner

**Affiliations:** The Warren Alpert Medical School Brown University, Rhode Island Hospital, Department Of Psychiatry, Providence, United States of America

## Abstract

There has been a significant increase in the incidence of depression in countries around the world during the COVID-19 pandemic. Identified concerns include: loss of family members, fear of gettin sick, finances, decreased social connections, deteriorating relationships at home and decreased ability to practise previously helpful coping skills. Family/couples intevrentions alone or in combination with individual therapy and/or pharmacotherapy have been shown to be helpful in diminishing symptoms of depression and in improving family functioning. This presentation will review evidence examining the effctiveness of family/couples therapy in treating adults with depression and outline therapy processes that have been shown to be effective.

**Disclosure:**

No significant relationships.

